# Supplementary material for: Caldera resurgence during the 2018 eruption of Sierra Negra volcano, Galápagos Islands
Source: Nat Commun. 2021 Mar 2;12:1397. doi: 10.1038/s41467-021-21596-4 (PMC7925514; doi:10.1038/s41467-021-21596-4)
Supplement: Supplementary file 1 — Supplementary Information [file 41467_2021_21596_MOESM1_ESM.pdf]

Supplementary Materials for

Caldera resurgence during the 2018 eruption cycle of Sierra Negra volcano, Galápagos Islands

Andrew F. Bell[1]*, Peter C. LaFemina[2], Mario Ruiz[3], Falk Amelung[4], Marco Bagnardi[5], Christopher J. Bean[6], Benjamin Bernard[3], Cynthia Ebinger[7], Matthew Gleeson[8], James Grannell[6], Stephen Hernandez[3], Machel Higgins[2], Céline Liorzou[9], Paul Lundgren[10], Nate Meier[2], Martin Möllhoff[6], Sarah-Jaye Oliva[7], Andres Gorki Ruiz[2], and Michael J. Stock[11]

Correspondence to: a.bell@ed.ac.uk

Supplementary Figures 1 - 4

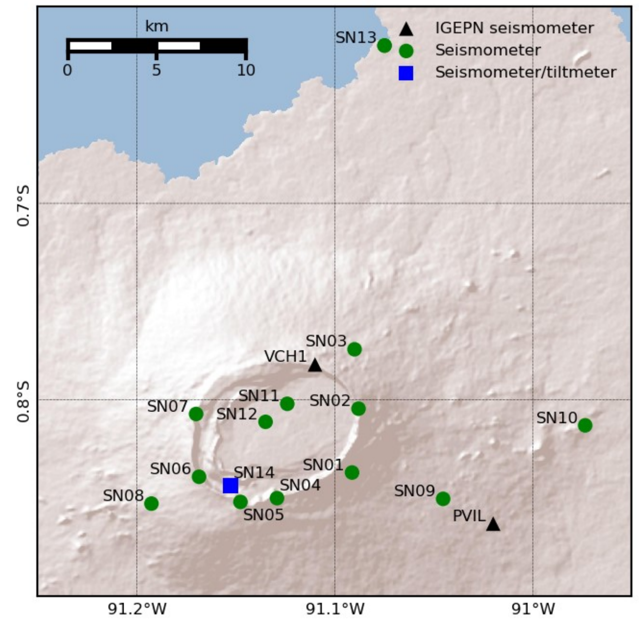

Supplementary Fig. 1. Location of broadband seismometers and tiltmeter deployed on Sierra Negra as part of the IGUANA network in April 2018, and permanent stations of the IGEPN monitoring network operational since 2012.

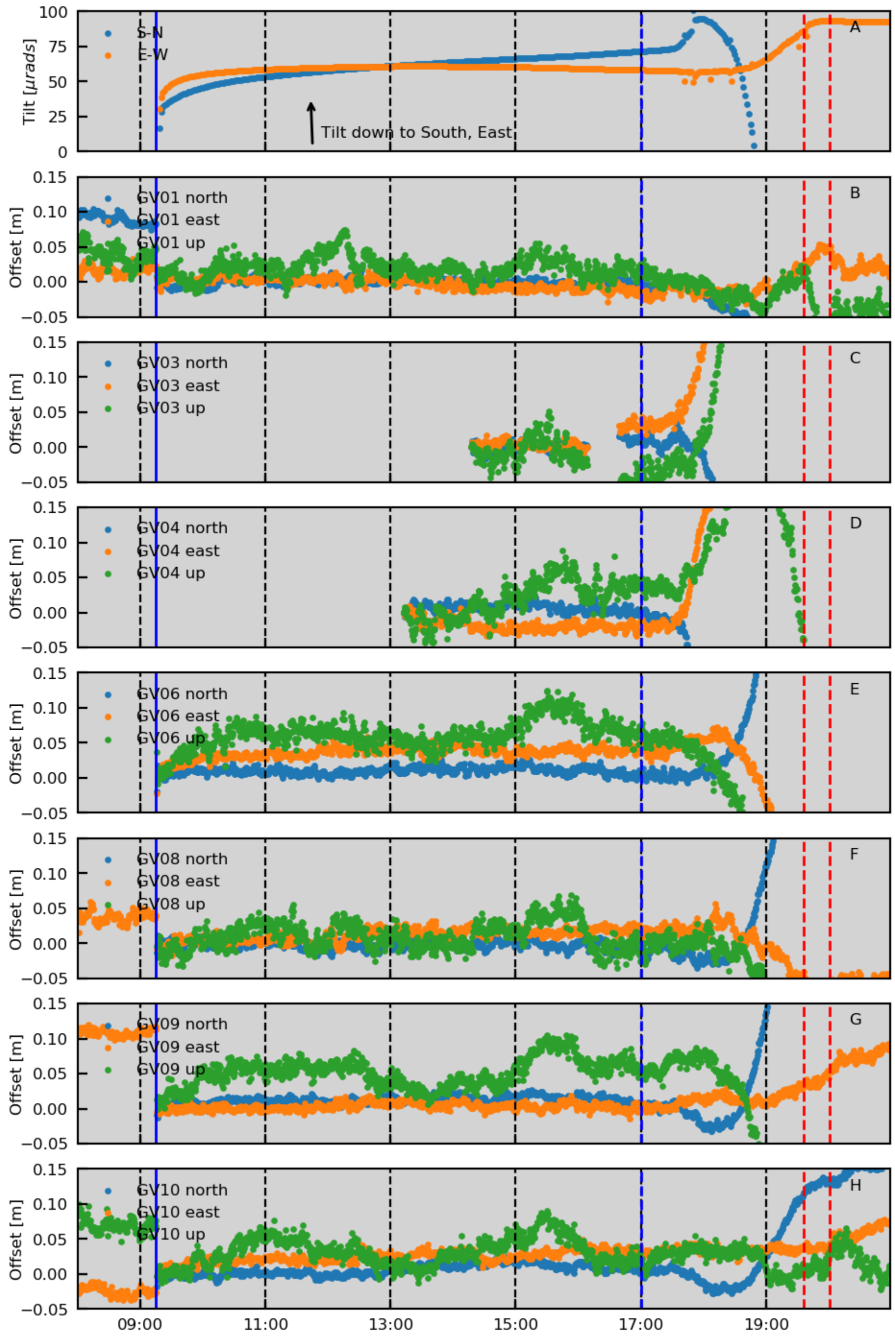

Supplementary Fig. 2. Deformation between 08:00 and 21:00 at Sierra Negra on 26 June 2018, with y-axis limits chosen to show low amplitude variations between the Mw 5.4 earthquake and onset of shallow magma migration. (A) Tilt recorded at station SN14. (B)-(H) Deformation recorded at cGPS stations. All tilts and displacements are relative to absolute value at 09:17. Mw5.4 earthquake occurred at 09:15 (vertical blue solid line). Two tremor pulses marking the onset of eruption indicated by vertical red dashed lines, and onset of seismic swarm at 17:00 indicated by blue dashed line.

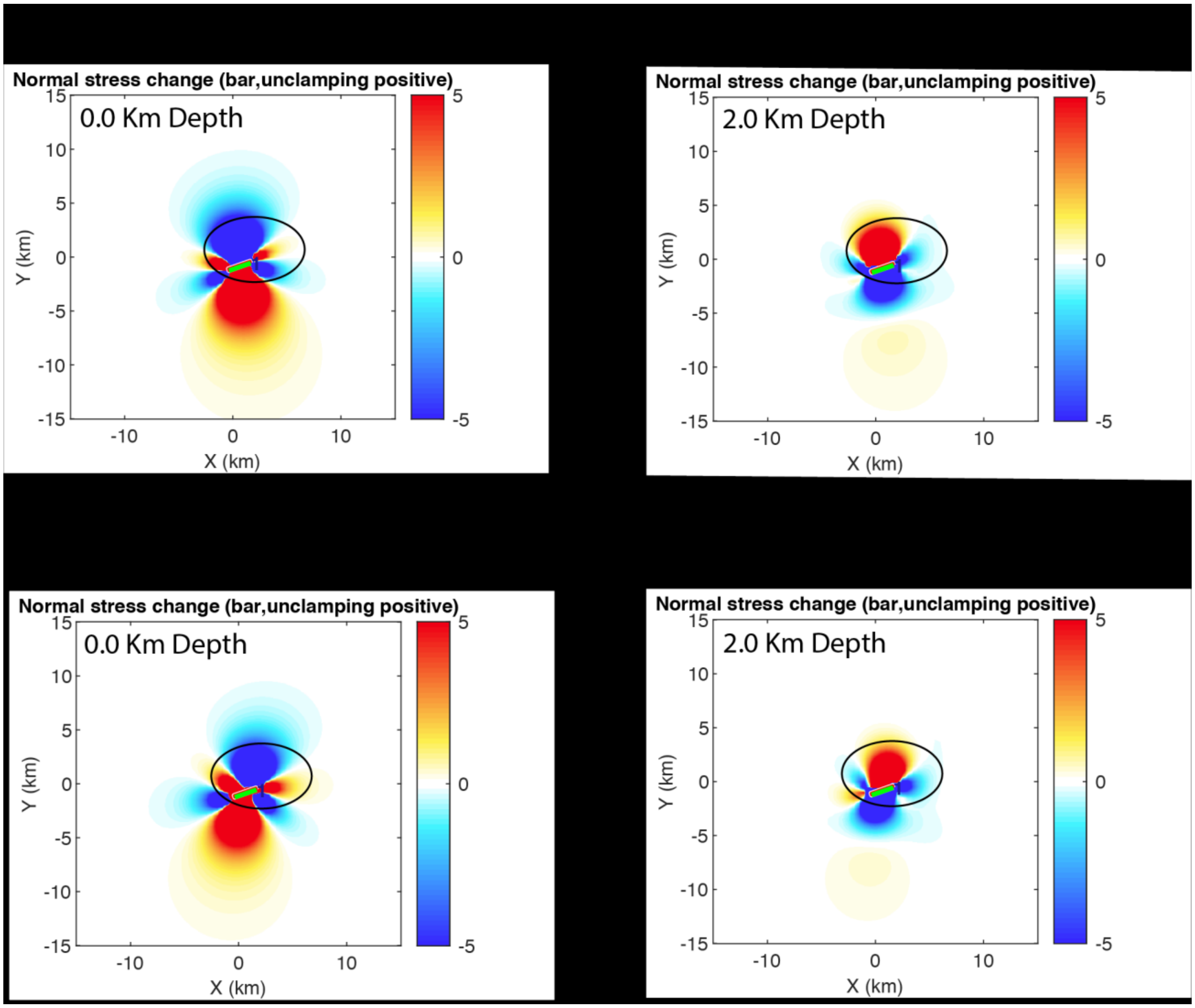

Supplementary Fig. 3. Normal stress change calculations for the 9:15 UTC 26 June 2018 earthquake. The strike of the USGS NEIC focal mechanism solution was used (strike 250º), but we used a dip of 85º, and rake of 90º. Normal stress change was calculated at 0.0 km and 2.0 km depths on faults with strikes of 90º and 120º, dip of 85º and rake of -90º.

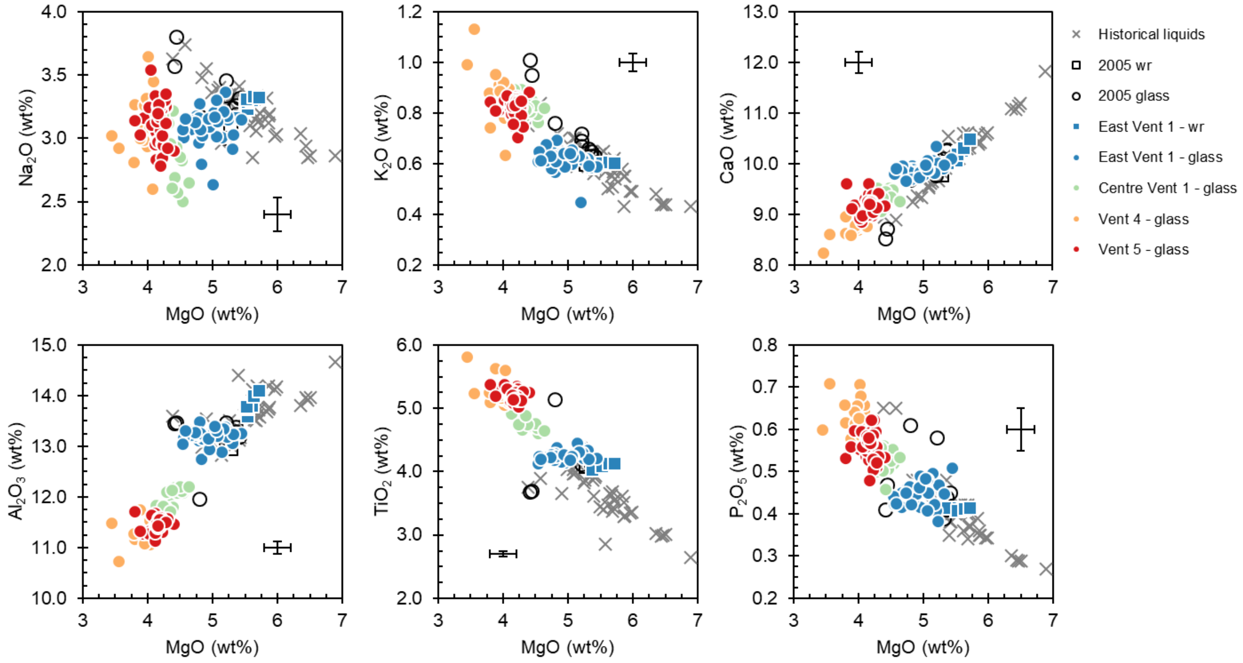

Supplementary Fig. 4.

Major and minor element compositions of whole-rock (wr; squares) and glass (circles) samples erupted at Sierra Negra. Colored points show material produced from different vents (at different times) during the 2018 eruption, black points show material from the 2005 eruption and grey points show material from older Sierra Negra eruptions (see legend). Error bars show representative 1σ uncertainties on our EPMA data. Data from pre-2018 eruptions are from[1–5].

Supplementary references

1. Geist, D. J. *et al.* The 2005 eruption of Sierra Negra volcano, Galápagos, Ecuador. *Bull. Volcanol.* **70**, 655–673 (2008).
2. Handley, H. K., Turner, S., Berlo, K., Beier, C. & Saal, A. E. Insights into the Galápagos plume from uranium-series isotopes of recently erupted basalts. *Geochemistry, Geophys. Geosystems* **12**, n/a-n/a (2011).
3. Kurz, M. D. & Geist, D. J. Dynamics of the Galapagos hotspot from helium isotope geochemistry. *Geochim. Cosmochim. Acta* **63**, 4139–4156 (1999).
4. SAAL, A. *et al.* The role of lithospheric gabbros on the composition of Galapagos lavas. *Earth Planet. Sci. Lett.* **257**, 391–406 (2007).
5. White, W. M., McBirney, A. R. & Duncan, R. A. Petrology and geochemistry of the Galápagos Islands: Portrait of a pathological mantle plume. *J. Geophys. Res. Solid Earth* **98**, 19533–19563 (1993).
