# Supplementary material for: Caldera resurgence during the 2018 eruption of Sierra Negra volcano, Galápagos Islands
Source: Nat Commun. 2021 Mar 2;12:1397. doi: 10.1038/s41467-021-21596-4 (PMC7925514; doi:10.1038/s41467-021-21596-4)
Supplement: Supplementary file 3 — Description of Additional Supplementary Files [file 41467_2021_21596_MOESM3_ESM.pdf]

Description of Additional Supplementary Files

File name: Supplementary Movie 1
Description: Deformation and seismicity before and during the 2018 eruption of Sierra Negra (26/04/2018-31/08/2018). (A) Magnitudes of earthquakes located by the IGUANA network. (B) Uplift recorded at GV04. Red dashed line indicates time of eruption onset. (C) Temporal evolution of epicentres of earthquakes (symbols corresponding to those in (A)), and horizontal (black) and vertical (red) displacement vectors at cGPS stations (green stars)

File name: Supplementary Movie 2
Description: Deformation and seismicity during the onset of the 2018 eruption of Sierra Negra (26/04/2018-25/08/2018). (A) Magnitudes of earthquakes located by the IGUANA network. (B) Uplift recorded at GV04. Black dashed line indicates time of Mw5.4 earthquake, and red dashed line indicates time of eruption onset. (C) Temporal evolution of epicentres of earthquakes (symbols corresponding to those in (A)), and horizontal (black) and vertical (red) displacement vectors at cGPS stations (green stars)
